# Liver Disease, Liver Fibrosis, and the Invasive-Management Gap in Acute Myocardial Infarction: A Single-Center Cohort with Dual ICD and FIB-4 Stratification

**DOI:** 10.3390/jcdd13090408

**Published:** 2026-08-24

**Authors:** Arun Gajan Pradeep, Muhammad Abdurrahman Butt, Kaiyu Jia, Bishoy Beshay, Jessica Meng, Saif Yasin, Esther Pearce, Thomas Gut

**Affiliations:** Department of Medicine, Staten Island University Hospital, Northwell Health, Staten Island, NY 10305, USA

**Keywords:** acute myocardial infarction, cirrhosis, liver fibrosis, FIB-4, invasive management, outcomes

## Abstract

Patients with chronic liver disease are systematically excluded from acute myocardial infarction (AMI) trials, and prior real-world data rely on administrative coding alone. Whether ICD-coded liver disease and laboratory-defined liver fibrosis identify the same patients, and whether they predict the same outcomes, is unknown. We conducted a single-center retrospective cohort study of 1037 consecutive adults admitted with AMI (ICD-10 I21.x) to a tertiary New York center between November 2022 and December 2024. The primary exposure was ICD-defined advanced liver disease (cirrhosis, hepatic failure, or portal hypertension/decompensation; *n* = 102). The secondary, lab-based exposure was the Fibrosis-4 (FIB-4) index calculated from earliest admission AST, ALT, and platelet count (computable in 1031 patients, 99.4%), stratified as low (<1.45), indeterminate (1.45–3.25), or advanced (>3.25). Co-primary outcomes were invasive management (diagnostic angiography, percutaneous coronary intervention, or coronary artery bypass grafting) and in-hospital mortality. Multivariable logistic regression adjusted for age, sex, diabetes, chronic kidney disease, heart failure, and ST-elevation; the trend across FIB-4 tiers was assessed with the Cochran–Armitage test. Denominators throughout (including the 168/909 occult-fibrosis estimate) use the full exposure group as denominator under a missing-as-not-exposed convention; the four no-LD and two advanced-LD patients with missing FIB-4 are counted as non-advanced fibrosis for this calculation. Patients with ICD-defined advanced liver disease received invasive management less often (12.7% vs. 50.4%; adjusted odds ratio [aOR] 0.17, 95% CI 0.09–0.32) and died in hospital more often (43.1% vs. 7.9%; aOR 8.22, 95% CI 5.02–13.46) than patients without coded liver disease. Outcomes worsened monotonically across FIB-4 tiers (mortality 4.4% → 9.2% → 27.5%; invasive management 55.2% → 45.6% → 33.5%; both *p* < 0.001 by Cochran–Armitage trend test). Critically, 168 of 909 patients with no coded liver disease (18.5%) had FIB-4 > 3.25, representing a substantial population of unrecognized advanced fibrosis missed by clinical coding. Coded liver disease identifies a small, severely affected subgroup with markedly lower rates of invasive management and 6- to 8-fold higher mortality after AMI. Routine FIB-4 calculation, a free, three-variable lab score, identifies a much larger population with occult advanced fibrosis and graded excess risk that ICD codes miss entirely. Pending prospective validation, FIB-4 may serve as a low-cost adjunct to bedside risk stratification in AMI care.

## 1. Clinical Perspective

**What Is New?**

- In a single-center cohort of 1037 consecutive AMI admissions, patients with ICD-coded advanced liver disease underwent invasive management at one-quarter the rate of patients without coded liver disease (12.7% vs. 50.4%) and had 8-fold higher adjusted in-hospital mortality (aOR 8.22; 95% CI 5.02–13.46).
- **Routine admission Fibrosis-4 (FIB-4) calculation, which was computable in 99.4% of the cohort from age, AST, ALT, and platelet count, identified an additional 168 patients with advanced fibrosis (FIB-4 > 3.25) but no coded liver disease, representing 18.5% of patients without any administrative liver disease label.**
- Outcomes worsened monotonically across FIB-4 tiers (in-hospital mortality 4.4% → 9.2% → 27.5%), with mortality risk approximately 5-fold higher in the advanced-fibrosis tier, independent of coded liver disease.

**What Are the Clinical Implications?**

- The diagnostic label of liver disease, separate from biological severity, **is associated with substantial differences in invasive AMI management. The gap between coded and uncoded-but-FIB-4-advanced patients is large enough to warrant clinician awareness during management decisions.**
- **FIB-4, a free, three-variable score derived from routine admission labs, could function as a near-universal bedside risk-stratification tool in AMI care by identifying a large at-risk population currently invisible to administrative coding.**
- Prospective evaluation of risk-stratified invasive strategies in patients with advanced fibrosis is warranted, particularly given that legitimate concerns about bleeding, contrast-induced AKI, and goals of care **may justify some, but not all, of the observed disparity.**

## 2. Introduction

Acute myocardial infarction (AMI) remains a leading cause of in-hospital mortality, with management algorithms increasingly standardized around early invasive strategies for both ST-elevation (STEMI) and non-ST-elevation (NSTEMI) presentations [1]. Patients with concomitant liver disease, however, occupy a poorly characterized clinical space: they are routinely excluded from randomized trials of antithrombotic therapy and revascularization, leaving clinicians without high-quality evidence to balance ischemic, bleeding, and contrast-related risks. Cirrhosis confers coagulopathy and thrombocytopenia, which elevate procedural bleeding risk, while simultaneously increasing the likelihood of contrast-induced acute kidney injury and decompensation during the peri-MI period. Importantly, this coagulopathy does not equate to protection from thrombosis: liver disease produces a simultaneous decline in both procoagulant and anticoagulant factors, a state of rebalanced but unstable hemostasis in which thrombotic complications occur alongside bleeding risk [2]. The pathophysiologic basis for adverse cardiovascular outcomes in advanced liver disease involves bidirectional cardiohepatic interactions, including cirrhotic cardiomyopathy, altered systemic hemodynamics, and hepatic congestion from right-heart dysfunction [3], which operate independently of traditional atherosclerotic risk factors.

Prior administrative-database studies have established that patients with ICD-coded cirrhosis presenting with AMI receive coronary angiography and revascularization at lower rates than non-cirrhotic patients (Hillerson et al., J Invasive Cardiol 2019; *n* = 3135 cirrhotic patients from the National Inpatient Sample) [4]. More recently, single-center registries have evaluated the Fibrosis-4 (FIB-4) index, a simple non-invasive score derived from age, AST, ALT, and platelet count, as a prognostic tool in acute coronary syndromes (Biccirè et al., Clin Res Cardiol 2023; Gorur et al., Intern Emerg Med 2025) [5,6]. These two bodies of literature, however, have remained largely disconnected: cirrhosis cohorts rarely report FIB-4, and FIB-4 cohorts are typically restricted to patients selected for angiography and do not capture the broader population of AMI admissions whose management decisions are influenced by clinician-perceived liver disease.

We hypothesized that ICD-defined liver disease and FIB-4-defined liver fibrosis would identify partly overlapping but clinically distinct populations within an unselected AMI cohort, with implications both for risk stratification and for understanding the apparent under-utilization of invasive management. We further hypothesized that a substantial fraction of AMI patients without any coded liver disease would harbor occult advanced fibrosis identifiable on routine admission labs. To test these hypotheses, we conducted a dual-exposure analysis of all consecutive AMI admissions to a tertiary New York hospital.

## 3. Methods

### 3.1. Study Design and Population

We performed a single-center retrospective cohort study of consecutive adult (≥18 years) patients admitted to Staten Island University Hospital (SIUH) North, a tertiary academic center within Northwell Health, with at least one diagnosis of acute myocardial infarction (International Classification of Diseases, 10th Revision [ICD-10] codes I21.x) recorded during the index encounter between November 2022 and December 2024. STEMI was defined by ICD-10 codes I21.0–I21.2, I21.3, or I22.1 (subsequent STEMI); NSTEMI was defined by I21.4 or I22.2 (subsequent NSTEMI). The 46 patients carrying codes from both ranges were assigned to STEMI under a STEMI-precedence rule, yielding a mutually exclusive STEMI/NSTEMI variable (STEMI *n* = 292, 28.2%; NSTEMI *n* = 745, 71.8%). Type 2 MI was identified separately by code I21.A1 and treated as a non-exclusive overlay that could co-occur with either STEMI or NSTEMI (*n* = 94, 9.1%: 5 STEMI, 89 NSTEMI) rather than as a third mutually exclusive category; codes for other or unspecified MI types (I21.9, I21.A9, I21.B) were not used to define subtype. The study was approved by the Northwell Health Institutional Review Board (Protocol 25-1044) and granted exemption under 45 CFR 46.104(d)(4)(iii) (secondary research uses of identifiable health information) with a full waiver of HIPAA authorization, on the basis that the research involves no more than minimal risk to privacy, could not practicably be conducted without the waiver, and could not be conducted without access to protected health information. A waiver of informed consent was granted accordingly. Reporting follows the STROBE guideline for cohort studies [7].

### 3.2. Exposure Definitions

The primary exposure was ICD-defined advanced liver disease (LD), operationalized as the presence at index admission or during prior encounters of any ICD-10 code for cirrhosis (K74.60, K74.69, K70.30, K70.31, K74.3–K74.5), hepatic failure (K72.0x, K72.1x, K72.9x), or portal hypertension/decompensation (K76.6, R18.0, R18.8, I85.0x, I85.1x, I86.4, K65.2, K76.7, G93.4x). Patients without any of these codes but carrying codes for fatty liver disease (K76.0, K75.81), chronic viral hepatitis (B18.x), or other unspecified chronic liver disease (K75.4, K76.1, K76.82, K76.89, K76.9) were classified as “Other chronic liver disease” (Other CLD); the remainder were classified as “No liver disease” (No LD). Strict cirrhosis ICD codes (K74.60, K74.69, K70.30, K70.31) appeared in only 9 patients in our source data; an additional 93 patients carried codes for hepatic failure or portal hypertension/decompensation without a documented cirrhosis code, consistent with well-documented under-coding of cirrhosis in administrative datasets [8]. The composite advanced-LD definition (*n* = 102) was therefore used as the primary exposure.

The secondary, lab-based exposure was the Fibrosis-4 index (FIB-4), calculated as (Age × AST)/(Platelets [×10^9^/L] × √ALT [IU/L]) using the earliest available values during the index admission [9]. Patients were stratified into low (<1.45), indeterminate (1.45–3.25), and advanced (>3.25) categories per established cutoffs. The Aspartate Aminotransferase-to-Platelet Ratio Index (APRI) was calculated for descriptive comparison.

### 3.3. Outcomes

Co-primary outcomes were (a) invasive management, defined as the receipt of diagnostic coronary angiography, percutaneous coronary intervention (PCI), or coronary artery bypass grafting (CABG) during the index admission (Current Procedural Terminology [CPT] 92920–92944, 93454–93461, 33510–33536) and (b) in-hospital all-cause mortality (death during the index admission or discharge to hospice). Secondary outcomes were acute kidney injury (AKI; ICD-10 N17.x), cardiogenic shock (R57.0), cardiac arrest (I46.2/I46.9), gastrointestinal bleeding (K92.0–2), sepsis (A41.x), acute respiratory failure (J96.0x), and length of stay.

### 3.4. Statistical Analysis

Continuous variables are reported as mean ± SD or median (IQR) and compared with Welch’s *t*-test or the Mann–Whitney U test, as appropriate; categorical variables are reported as *n* (%) and compared with Pearson χ^2^ or Fisher’s exact test when expected counts were <5. Trend across the three ordered FIB-4 tiers was assessed using the Cochran–Armitage test for binary outcomes. Multivariable logistic regression models were fit by iteratively reweighted least squares (Newton–Raphson with Fisher scoring) to estimate adjusted odds ratios (aORs) for each exposure, restricting the ICD-based comparison to advanced LD versus no LD (*n* = 1011); the 26 patients with Other CLD were retained in descriptive analyses but excluded from regression models. Models were adjusted for age, sex, diabetes mellitus, chronic kidney disease (CKD), heart failure, and ST-elevation. The primary models estimate the total association between liver disease and each outcome. Because cardiogenic shock may lie on the pathway between liver disease and death rather than being a fixed pre-admission characteristic, and because our source data did not permit reliable separation of admission-time shock from shock developing during the encounter, shock was not included in the primary models; models additionally adjusting for it are reported separately (Table S3) to describe the portion of the association not accounted for by acute presentation severity. For mortality, we additionally fit a secondary model that included invasive management as a covariate, to describe the extent to which the mortality association attenuates when adjustment is made for revascularization receipt. We did not perform a formal causal mediation analysis (e.g., natural direct/indirect effect decomposition with bootstrap confidence intervals); the with/without-invasive-management comparison is descriptive and should not be interpreted as identifying a controlled direct effect. Pre-specified sensitivity analyses included (1) restriction to a per-protocol cohort excluding patients with prior MI, prior PCI, prior CABG, or liver transplant codes and (2) substitution of the broader “any chronic liver disease” definition. The shock-adjusted models described above are reported as a post hoc sensitivity analysis (Table S3). All tests were two-sided with α = 0.05.

## 4. Results

### 4.1. Cohort Characteristics

Of 1037 patients meeting the inclusion criteria during the study period, 102 (9.8%) carried codes for advanced liver disease, 26 (2.5%) carried codes only for other chronic liver disease, and 909 (87.7%) had no coded liver disease. Patients with advanced liver disease were older than those without coded liver disease (mean 73.5 ± 13.1 vs. 68.4 ± 13.3 years, *p* < 0.001) and less frequently male (59.8% vs. 66.8%); racial composition was similar (70.6% vs. 70.2% White; 8.8% vs. 6.1% African American/Black), although Hispanic ethnicity was less common in the advanced liver disease group (2.9% vs. 7.8%). The advanced liver disease group carried substantially higher comorbid burdens, including diabetes mellitus (57.8% vs. 44.4%), heart failure (61.8% vs. 39.9%), and chronic kidney disease (44.1% vs. 25.6%); hypertension prevalence was similar (86.3% vs. 83.9%) (Table 1). NSTEMI was the dominant MI subtype in both groups and was somewhat more common in the advanced liver disease cohort, although this difference did not reach statistical significance (78.4% vs. 70.8% by mutually exclusive primary MI classification, p = 0.11); STEMI accounted for 21.6% of advanced liver disease admissions vs. 29.2% of patients without coded liver disease.

FIB-4 stratification was feasible in 1031 of 1037 patients (99.4%): 388 (37.6%) had low FIB-4, 425 (41.2%) indeterminate, and 218 (21.1%) advanced FIB-4 (>3.25). Notably, of 909 patients without coded liver disease, 168 (18.5%) had FIB-4 > 3.25, indicating substantial occult advanced fibrosis missed by clinical coding (Table S1). Concordant findings were observed with the APRI score: the median APRI was 0.49 (IQR 0.27–1.04) in the advanced-LD group versus 0.29 (0.19–0.49) in patients with no coded liver disease (*p* < 0.001), and APRI ≥ 1.0 (the threshold suggestive of significant fibrosis) was present in 28.0% (28/100) of advanced-LD patients versus 8.1% (73/905) of patients without coded liver disease.

### 4.2. Invasive Management

**Figure 1:**
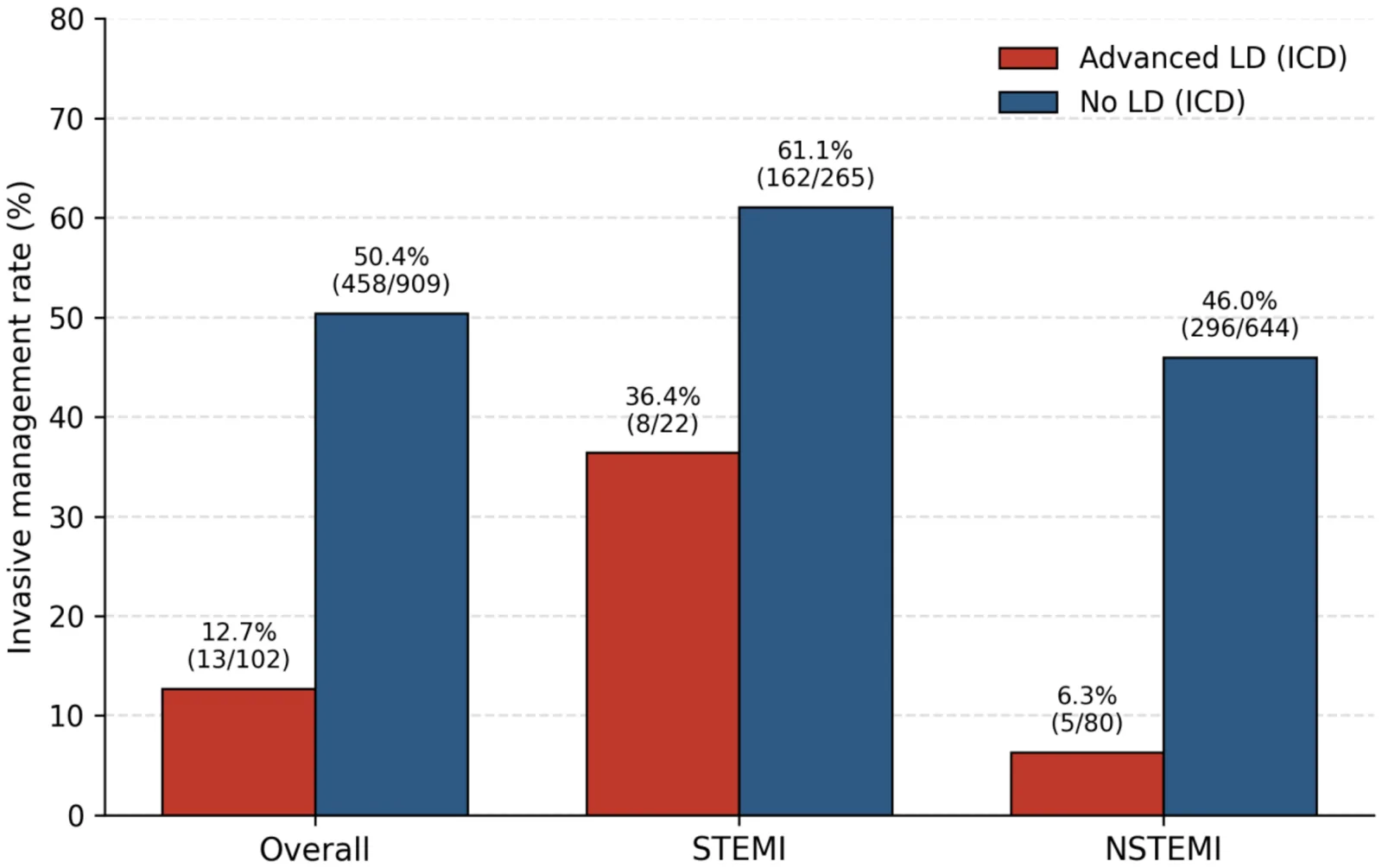
Invasive management rate (diagnostic angiography, PCI, or CABG) by MI subtype and ICD-defined liver disease status. Numerators and denominators are shown above each bar. Within NSTEMI, the absolute disparity is largest (6.3% vs. 46.0%), suggesting that the disparity in invasive care rates by liver disease status cannot be explained by primary-PCI logistics alone.

Patients with ICD-defined advanced liver disease underwent invasive management in 13 of 102 (12.7%) cases, compared with 458 of 909 (50.4%) patients without coded liver disease (*p* < 0.001; Table 2). The disparity persisted within MI subtypes, but it was strikingly larger in NSTEMI (6.3% vs. 46.0%) than in STEMI (36.4% vs. 61.1%) (Figure 1). The STEMI subgroup comparison should be interpreted as exploratory and hypothesis-generating given the small advanced-LD STEMI denominator (*n* = 22). PCI was performed in 6.9% of advanced liver disease patients vs. 32.0% of patients without coded liver disease (*p* < 0.001). After multivariable adjustment for age, sex, diabetes, chronic kidney disease, heart failure, and ST-elevation, advanced liver disease was independently associated with markedly lower odds of invasive management (aOR 0.17, 95% CI 0.09–0.32, *p* < 0.001).

FIB-4 demonstrated a graded inverse association with invasive management: 55.2% (low), 45.6% (indeterminate), and 33.5% (advanced FIB-4) of patients in each tier received invasive management (Cochran–Armitage Z = −5.14, *p* < 0.001 for trend). The fully adjusted aOR for advanced FIB-4 versus low FIB-4 was 0.62 (95% CI 0.42–0.92, *p* = 0.019).

### 4.3. In-Hospital Mortality

**Figure 2:**
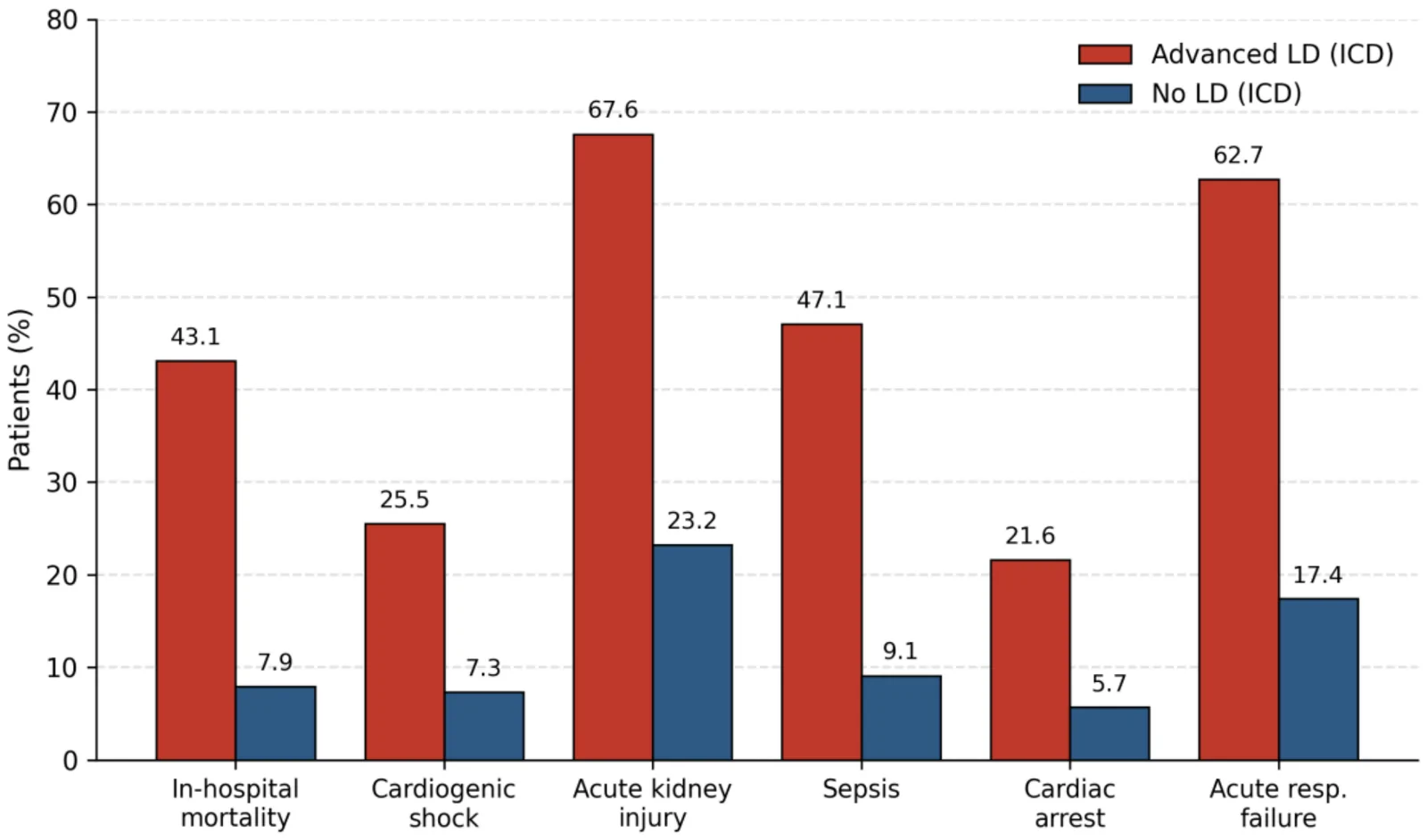
In-hospital outcomes by ICD-defined liver disease status. All differences shown are statistically significant at *p* < 0.001 (χ^2^ or Fisher’s exact test, as appropriate).

**Figure 3:**
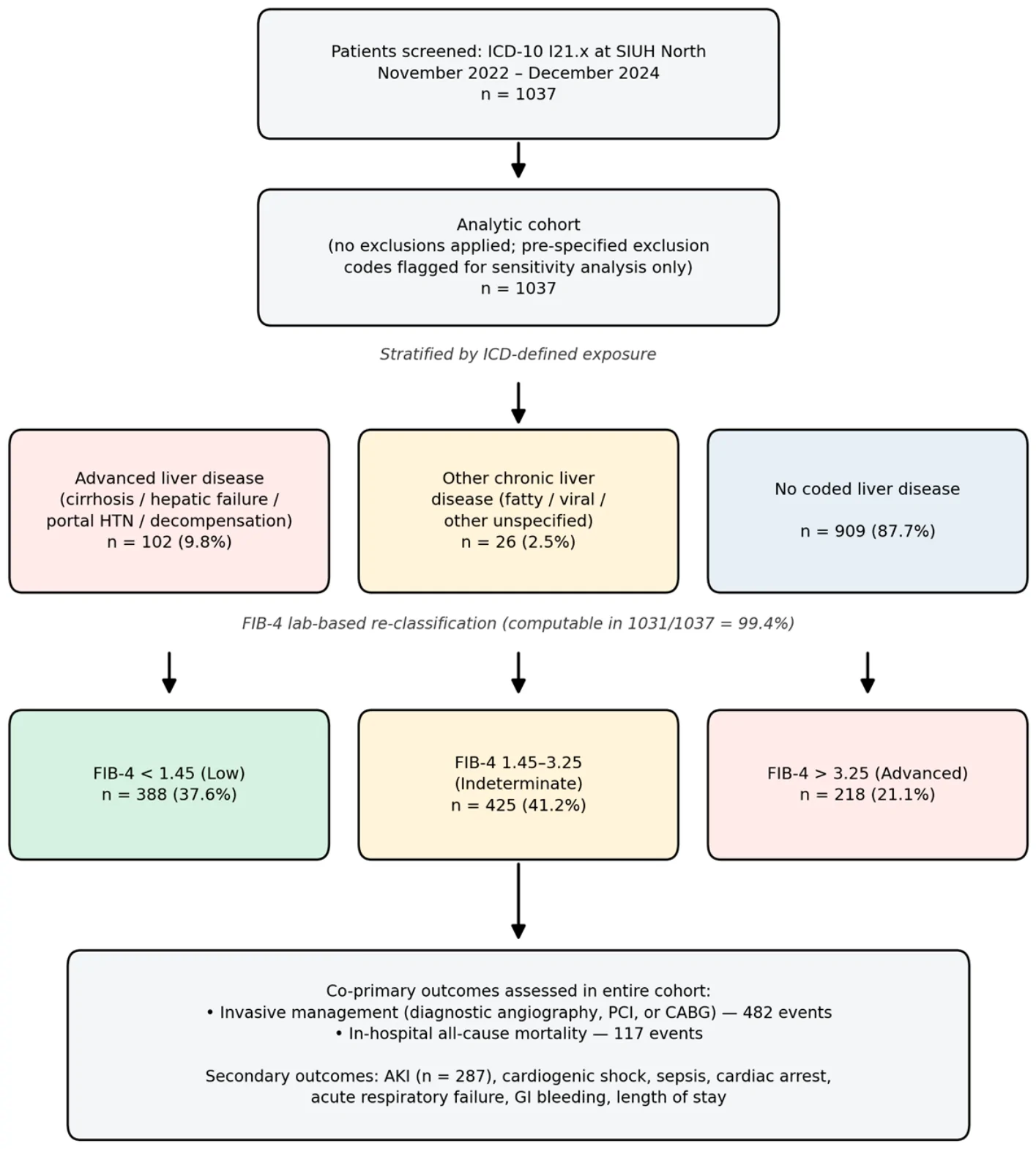
STROBE-style cohort flow diagram. The analytic cohort includes all 1037 consecutive AMI admissions; the 375 patients carrying any pre-specified exclusion code (prior MI, prior PCI, prior CABG, or liver transplant) were retained in the primary analysis and excluded only in the sensitivity analysis (S1). FIB-4 was computable in 1031 of 1037 patients (99.4%); the lower row therefore includes only the 1031 patients with computable FIB-4.

**Figure 4:**
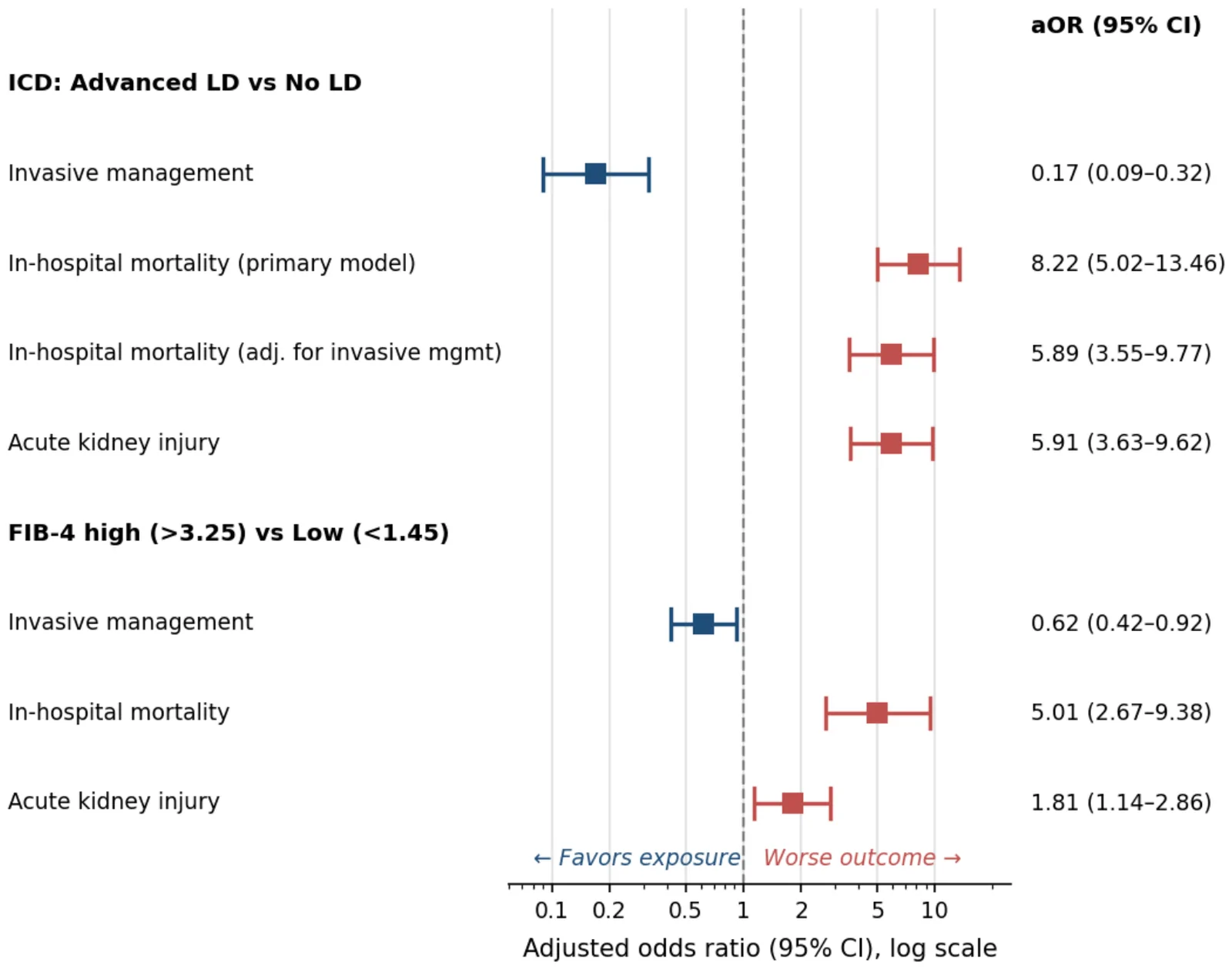
Forest plot of adjusted odds ratios for primary outcomes by ICD-defined and FIB-4-defined liver- disease exposures. Reference categories: no LD (ICD models) and low FIB-4 (<1.45). The primary mortality model adjusts for age, sex, diabetes, CKD, heart failure, and ST-elevation; the “adj. for invasive mgmt” row additionally includes invasive management as a covariate (descriptive only; not a formal mediation analysis). Squares represent point estimates; horizontal lines represent 95% confidence intervals. The vertical dashed line at OR = 1 represents no effect.

Forty-four of 102 patients (43.1%) with advanced liver disease died during the index admission, compared with 72 of 909 (7.9%) patients without coded liver disease (*p* < 0.001; Table 3, Figure 2). Group sizes and FIB-4 strata underlying all outcome analyses are shown in the cohort flow diagram (Figure 3). The adjusted odds ratio from the primary multivariable model was 8.22 (95% CI 5.02–13.46, *p* < 0.001); a secondary model additionally adjusted for invasive management yielded an aOR of 5.89 (95% CI 3.55–9.77, *p* < 0.001) (Table 4, Figure 4). FIB-4 again demonstrated a monotonic gradient: in-hospital mortality was 4.4%, 9.2%, and 27.5% across the low, indeterminate, and advanced tiers, respectively (Cochran–Armitage Z = 8.18, *p* < 0.001 for trend), with adjusted odds of mortality 5.01-fold higher (95% CI 2.67–9.38) in the advanced FIB-4 group versus low (Figure 4). In a post hoc sensitivity analysis additionally adjusting for cardiogenic shock, the mortality association attenuated modestly but remained large and highly significant (advanced LD vs. no LD: primary aOR 6.77, 95% CI 4.06–11.29; with invasive management additionally included, aOR 4.85, 95% CI 2.85–8.24), as did the AKI association (aOR 5.09, 95% CI 3.09–8.36; Table S3). These shock-adjusted estimates describe the portion of the association not accounted for by acute presentation severity; the primary models, which estimate the total association, remain the principal reference, and the qualitative conclusions are unchanged either way. To further quantify the robustness of the primary mortality association to unmeasured confounding, we calculated the E-value [10]: an unmeasured confounder would need to be associated with both advanced liver disease and mortality by a risk ratio of at least 9.9-fold each, above and beyond the measured covariates, to fully explain away the point estimate (E-value 7.1 at the limit of the 95% CI closer to the null).

### 4.4. Secondary Outcomes

Acute kidney injury occurred in 67.6% of advanced liver disease patients vs. 23.2% of patients without coded liver disease (aOR 5.91, 95% CI 3.63–9.62, *p* < 0.001; Figure 4), with an analogous monotonic gradient across FIB-4 tiers (19.3% → 29.4% → 39.4%; Cochran–Armitage Z = 5.40, *p* < 0.001 for trend; aOR 1.81, 95% CI 1.14–2.86, Figure 4). Median length of stay was 12.2 days (IQR 5.6–20.5) in patients with advanced liver disease versus 3.9 days (IQR 2.0–7.8) in those without coded liver disease (*p* < 0.001 by Mann–Whitney U). Cardiogenic shock (5.9% → 8.5% → 16.5% across FIB-4 tiers; *p* < 0.001 for trend), sepsis (8.5% → 11.8% → 24.3%; *p* < 0.001), cardiac arrest (3.6% → 5.2% → 17.9%; *p* < 0.001), and acute respiratory failure (14.9% → 20.7% → 35.8%; *p* < 0.001) all showed graded increases across FIB-4 tiers (Table 5) and were each significantly more frequent in the ICD-defined advanced liver disease group (Table 3, Figure 2).

### 4.5. Sensitivity Analyses

Findings were robust in the per-protocol cohort excluding 375 patients with prior MI, prior PCI, prior CABG, or liver transplant codes: invasive management rates were 13.2% (advanced LD) vs. 52.7% (no LD); in-hospital mortality was 44.1% vs. 8.3% (Table S2). The broader “any chronic liver disease” definition (*n* = 128 vs. 909) preserved direction and significance with attenuated magnitude (mortality 35.2% vs. 7.9%; invasive management 18.8% vs. 50.4%). We additionally assessed the sensitivity of the occult-fibrosis estimate (168/909, 18.5%) to the missing-as-not-advanced-fibrosis convention applied to the six patients with unmeasured FIB-4: reclassifying all four missing no-LD patients as advanced fibrosis yielded 172/909 (18.9%), while a complete-case approach excluding them yielded 168/905 (18.6%); the estimate is stable to within 0.4 percentage points under either assumption.

## 5. Discussion

In a contemporary single-center cohort of 1037 consecutive AMI admissions, we found that patients with ICD-defined advanced liver disease underwent invasive management at one-quarter the rate of patients without coded liver disease, with a more than eight-fold higher odds of in-hospital mortality after adjustment. Routine admission FIB-4 stratification, feasible in essentially the entire cohort, revealed a graded relationship between hepatic fibrosis severity and adverse outcomes that paralleled, but was distinct from, the ICD-coded relationship. Most strikingly, nearly one in five patients without any coded liver disease harbored FIB-4 evidence of advanced fibrosis, identifying a substantial unrecognized at-risk population invisible to administrative coding.

Our findings extend three prior lines of literature. First, we confirm and quantify the invasive-management gap previously reported by Hillerson and colleagues in a propensity-matched National Inpatient Sample analysis (2010–2014, *n* = 3135 cirrhotic vs. 3086 matched controls), where coronary angiography was performed in 51.4% of cirrhotic AMI patients vs. 63.9% of non-cirrhotic patients [4]. Our point estimate (12.7% vs. 50.4%) is more extreme, likely reflecting (i) a stricter ICD-defined exposure that captures clinically apparent decompensated disease rather than any cirrhosis code, (ii) the inclusion of patients managed without any cardiology consultation, and (iii) regional and more recent practice differences. Similar disparities have been reported in subsequent administrative-database analyses [11,12], including a recent NIS analysis (2016–2019) that found NAFLD was associated with 1.55-fold higher odds of AMI hospitalization and 1.96-fold higher mortality [13]; broader long-term post-MI outcomes, including substantial cumulative incidence of heart failure, renal failure, and severe bleeding, have been characterized in a recent population study of 56 million adults in England [14]. Second, our FIB-4 results are consistent with the prognostic associations reported by Biccirè et al., Cordero et al., and Gorur et al. in selected acute coronary syndrome–PCI populations [5,6,15], but apply for the first time to the broader unselected AMI admission population, including those who never reached the catheterization laboratory and whose risk would otherwise be invisible to FIB-4-in-PCI cohorts. Third, our identification of a sizable occult-fibrosis subgroup (*n* = 168) provides a quantitative anchor for the long-suspected coding bias in cirrhosis epidemiology and a tractable target for routine bedside screening. This phenomenon is especially relevant in the era of metabolic dysfunction-associated steatotic liver disease (MASLD), the new consensus nomenclature replacing non-alcoholic fatty liver disease (NAFLD) [16], which affects roughly 30% of the global adult population and is frequently undiagnosed until advanced fibrosis develops. Long-term outcomes after PCI in patients with end-stage liver disease have been described separately by Lu et al. [17], and a recent narrative review by Ang et al. summarizes the field [18].

The approximately 8-fold adjusted mortality gap is unlikely to be explained entirely by liver-related complications or by clinician-perceived bleeding risk leading to conservative management. Even after additionally adjusting for invasive management, advanced liver disease patients had approximately 6-fold higher mortality, suggesting substantial liver-disease-attributable risk that is not modifiable by changes in the revascularization strategy alone. Conversely, the magnitude of the invasive-management gap, particularly within NSTEMI, where the comparison is least confounded by the time-pressured logic of primary PCI, raises the possibility that some patients with advanced liver disease are being managed more conservatively than their cardiovascular risk alone would justify. We acknowledge that legitimate clinical concerns, including elevated procedural bleeding risk from coagulopathy and thrombocytopenia [19], contrast-induced AKI [20], and goals-of-care considerations in patients with advanced liver disease [21], may appropriately drive a portion of this gap. Notably, several procedural strategies, including default radial access (which reduced 30-day all-cause mortality from 2.1% to 1.6% [*p* = 0.012] and major bleeding from 2.7% to 1.5% [*p* < 0.001] versus femoral access in a 21,600-patient individual-patient-data meta-analysis of seven randomized trials [22]), ultra-low-volume contrast protocols, and bleeding-conscious antithrombotic regimens, can substantially mitigate the procedural risks that drive conservative management in this population. The current ACC/AHA acute coronary syndromes guideline [1] endorses these strategies as the standard of care, none of which are contraindicated by liver disease per se. Whether the residual disparity reflects under-treatment or appropriate risk-adjusted decision-making cannot be determined from observational data alone, and disentangling them will require prospective study.

Clinical implications are immediate and tractable. FIB-4 requires only age, AST, ALT, and platelet count, all routinely measured at AMI admission, and adds no incremental cost. Its incorporation into AMI risk-stratification pathways could identify the substantial occult-fibrosis population currently missed by ICD coding alone, prompting earlier hepatology consultation, contrast minimization, radial access preference, and individualized antithrombotic decision-making.

### 5.1. Limitations

This is a single-center retrospective study from one academic hospital in New York; generalizability to other regions, demographics, and practice patterns requires validation. Exposure ascertainment relied on ICD-10 coding and laboratory data; chart review for the Model for End-Stage Liver Disease (MELD) score, Child–Pugh class, and decompensation severity was beyond the scope. FIB-4 cutoffs were validated in chronic hepatitis populations [9], and acute MI may transiently elevate AST from cardiac muscle and alter platelet counts through acute-phase and consumptive effects, potentially overestimating fibrosis at admission; however, our use of earliest-encounter values is the standard approach and would bias toward the null rather than amplify the observed gradient. Outcomes were limited to in-hospital events; long-term follow-up was not available. The STEMI subgroup within the advanced liver disease cohort was small (*n* = 22) and the corresponding invasive-management comparison should be regarded as exploratory; replication in larger cohorts is needed. We did not perform a formal causal mediation analysis; the with/without-invasive-management mortality comparison is descriptive rather than a controlled direct effect, and should be interpreted as describing the extent of statistical attenuation rather than identifying a counterfactual quantity. Because cardiogenic shock may lie on the causal pathway between liver disease and death, and because its timing relative to admission could not be reliably established in our source data, it was excluded from the primary models, which estimate the total association; a sensitivity analysis additionally adjusting for shock is reported (Table S3) and did not materially alter the findings. Finally, residual confounding by frailty, hepatic synthetic function, malnutrition, alcohol use, coagulopathy, and operator-perceived bleeding risk cannot be excluded.

### 5.2. Conclusions

ICD-coded advanced liver disease and FIB-4-defined liver fibrosis identify partly overlapping but distinct populations of AMI patients at substantially elevated risk. The ICD-defined population is small, severely affected, and revascularized substantially less often; the FIB-4-defined population is much larger and includes a substantial occult-fibrosis subgroup invisible to administrative coding. Admission FIB-4 calculation, a free and near-universal score, may warrant consideration as an adjunct to risk stratification in AMI care pending prospective validation; trials are needed to determine whether targeted intensification of care can narrow the observed mortality gap.

## Figures and Tables

**Table 1:** Baseline demographics and comorbidities by exposure group.

Variable	Advanced LD (*n* = 102)	Other CLD (*n* = 26)	No LD (*n* = 909)	Total (*n* = 1037)	*p* *
***Demographics***					
Age, mean (SD), years	73.5 (13.1)	—	68.4 (13.3)	69.0 (13.3)	<0.001
Male sex, *n* (%)	61 (59.8)	—	607 (66.8)	690 (66.5)	0.16
White race, *n* (%)	72 (70.6)	—	638 (70.2)	728 (70.2)	0.93
Black race, *n* (%)	9 (8.8)	—	55 (6.1)	65 (6.3)	0.28
Hispanic ethnicity, *n* (%)	3 (2.9)	—	71 (7.8)	77 (7.4)	0.07
***Comorbidities***					
Hypertension	88 (86.3)	—	763 (83.9)	871 (84.0)	0.54
Diabetes mellitus	59 (57.8)	—	404 (44.4)	473 (45.6)	0.01
Chronic kidney disease	45 (44.1)	—	233 (25.6)	287 (27.7)	<0.001
End-stage renal disease	12 (11.8)	—	53 (5.8)	67 (6.5)	0.02
Heart failure	63 (61.8)	—	363 (39.9)	437 (42.1)	<0.001
Atrial fibrillation	40 (39.2)	—	224 (24.6)	270 (26.0)	0.001
Anemia	47 (46.1)	—	185 (20.4)	247 (23.8)	<0.001
Prior coronary artery disease	54 (52.9)	—	703 (77.3)	773 (74.5)	<0.001
***MI subtype***					
STEMI	22 (21.6)	—	265 (29.2)	292 (28.2)	0.11
NSTEMI	80 (78.4)	—	644 (70.8)	745 (71.8)	0.11
Type 2 MI	18 (17.6)	—	73 (8.0)	94 (9.1)	0.001

* Pearson χ^2^ for categorical variables; Welch *t*-test for continuous variables. Comparison is advanced LD vs. no LD; the Other CLD group (*n* = 26) is reported descriptively only and not included in the formal comparison. Percentages are within-column. Bold rows are grouping headers and contain no data.

**Table 2:** In-hospital management strategies by exposure group.

Variable	Advanced LD (*n* = 102)	No LD (*n* = 909)	Other CLD (*n* = 26)	*p* *
Diagnostic angiography (CPT 93454–93461)	12 (11.8)	434 (47.7)	—	<0.001
Percutaneous coronary intervention (CPT 92920–92944)	7 (6.9)	291 (32.0)	—	<0.001
Coronary artery bypass grafting (CPT 33510–33536)	3 (2.9)	57 (6.3)	—	0.18
Any invasive management (cath, PCI, or CABG)	13 (12.7)	458 (50.4)	—	<0.001
Intra-aortic balloon pump	6 (5.9)	19 (2.1)	—	0.03 †
Impella	3 (2.9)	7 (0.8)	—	0.07 †
Endotracheal intubation	13 (12.7)	22 (2.4)	—	<0.001 †
STEMI subgroup: invasive management	8/22 (36.4)	162/265 (61.1)	—	0.02
NSTEMI subgroup: invasive management	5/80 (6.3)	296/644 (46.0)	—	<0.001

Values are *n* (%). * χ^2^ test (or † Fisher’s exact test where any expected cell < 5) comparing advanced LD vs. no LD. The Other CLD group is omitted for clarity. Percentages for STEMI/NSTEMI subgroups are within the corresponding subtype.

**Table 3:** In-hospital outcomes and complications by exposure group.

Variable	Advanced LD (*n* = 102)	No LD (*n* = 909)	Other CLD (*n* = 26)	*p* *
In-hospital mortality, *n* (%)	44 (43.1)	72 (7.9)	—	<0.001
Length of stay, mean (days)	15.3	6.2	—	<0.001
Length of stay, median (IQR)	12.2 (5.6–20.5)	3.9 (2.0–7.8)	—	<0.001
Acute kidney injury	69 (67.6)	211 (23.2)	—	<0.001
Cardiogenic shock	26 (25.5)	66 (7.3)	—	<0.001
Cardiac arrest	22 (21.6)	52 (5.7)	—	<0.001
Gastrointestinal bleeding	8 (7.8)	26 (2.9)	—	0.02 †
Stroke	19 (18.6)	73 (8.0)	—	<0.001
Acute respiratory failure	64 (62.7)	158 (17.4)	—	<0.001
Sepsis	48 (47.1)	83 (9.1)	—	<0.001

Values are *n* (%) unless otherwise specified. * χ^2^ (or † Fisher’s exact); Welch t for means; Mann–Whitney U for medians.

**Table 4:** Multivariable logistic regression: Adjusted odds ratios for primary outcomes.

Outcome and Exposure	Events/N	aOR	95% CI	*p*
***Invasive management* (*n* = 1011; 471 *events*)**				
Advanced LD vs. No LD (ICD)	13/102 vs. 458/909	**0.17**	0.09–0.32	<0.001
Advanced FIB-4 vs. Low FIB-4	73/218 vs. 214/388	**0.62**	0.42–0.92	0.019
***In-hospital mortality, primary multivariable model* (*n* = 1011; 116 *events*)**				
Advanced LD vs. No LD (ICD)	44/102 vs. 72/909	**8.22**	5.02–13.46	<0.001
Advanced FIB-4 vs. Low FIB-4	60/218 vs. 17/388	**5.01**	2.67–9.38	<0.001
***In-hospital mortality, additionally adjusted for invasive management***				
Advanced LD vs. No LD (ICD)	44/102 vs. 72/909	**5.89**	3.55–9.77	<0.001
Advanced FIB-4 vs. Low FIB-4	60/218 vs. 17/388	**4.95**	2.60–9.44	<0.001
***Acute kidney injury* (*n* = 1011; 280 *events*)**				
Advanced LD vs. No LD (ICD)	69/102 vs. 211/909	**5.91**	3.63–9.62	<0.001
Advanced FIB-4 vs. Low FIB-4	86/218 vs. 75/388	**1.81**	1.14–2.86	0.012

All models are fit by iteratively reweighted least squares (Newton–Raphson with Fisher scoring). ICD-based models compare advanced LD (*n* = 102) vs. no LD (*n* = 909) and are fit on *n* = 1011; the 26 patients with Other CLD were retained in descriptive analyses but excluded from regression models. FIB-4 models compare advanced FIB-4 (>3.25) vs. low FIB-4 (<1.45). All models adjust for age, sex, diabetes, CKD, heart failure, and ST-elevation (AKI model omits ST-elevation). The primary models estimate the total association between liver disease and each outcome; cardiogenic shock, which may lie on the pathway between liver disease and death and whose timing could not be reliably established in our source data, was not included as a covariate. See Table S3 for shock-adjusted sensitivity analysis describing the residual association. For mortality, the primary multivariable model is reported alongside a secondary model that additionally includes invasive management as a covariate. The secondary model is descriptive of the extent to which the mortality association attenuates when adjustment is made for revascularization receipt; it does not constitute a formal mediation analysis (no bootstrap CIs, no exposure–mediator interaction) and the aOR should not be interpreted as a controlled direct effect. Bold rows are grouping headers and contain no data.

**Table 5:** FIB-4 distribution and outcomes by tier.

Variable	Low FIB-4 (<1.45) *n* = 388	Indeterminate (1.45–3.25) *n* = 425	Advanced FIB-4 (>3.25) *n* = 218
Distribution, *n* (%)	388 (37.6)	425 (41.2)	218 (21.1)
Invasive management, *n* (%)	214 (55.2)	194 (45.6)	73 (33.5)
Percutaneous coronary intervention, *n* (%)	142 (36.6)	119 (28.0)	43 (19.7)
In-hospital mortality, *n* (%)	17 (4.4)	39 (9.2)	60 (27.5)
Acute kidney injury, *n* (%)	75 (19.3)	125 (29.4)	86 (39.4)
Cardiogenic shock, *n* (%)	23 (5.9)	36 (8.5)	36 (16.5)
Sepsis, *n* (%)	33 (8.5)	50 (11.8)	53 (24.3)
Cardiac arrest, *n* (%)	14 (3.6)	22 (5.2)	39 (17.9)
Acute respiratory failure, *n* (%)	58 (14.9)	88 (20.7)	78 (35.8)
Length of stay, median (days)	3.7	4.3	5.7

FIB-4 = (Age × AST)/(Platelets [×10^9^/L] × √ALT [IU/L]), using the earliest values during the index admission. *p* < 0.001 by Cochran–Armitage trend test for invasive management, in-hospital mortality, AKI, cardiogenic shock, sepsis, cardiac arrest, and acute respiratory failure (Z statistics: −5.14, 8.18, 5.40, 4.12, 5.21, 5.98, 5.74, respectively).

## Data Availability

Deidentified data may be made available to qualified investigators upon reasonable request to the corresponding author and with appropriate institutional approvals.

## Supplementary Materials

The following supporting information can be downloaded at https://www.mdpi.com/article/10.3390/jcdd13090408/s1: Table S1: FIB-4 tier × ICD exposure cross-tabulation; Table S2: Sensitivity analyses; Table S3: Sensitivity analysis: mortality and AKI models additionally adjusted for cardiogenic shock (ICD-based exposure, *n* = 1011).
