# Supplementary material for: Liver Disease, Liver Fibrosis, and the Invasive-Management Gap in Acute Myocardial Infarction: A Single-Center Cohort with Dual ICD and FIB-4 Stratification
Source: J Cardiovasc Dev Dis. 2026 Aug 24;13(9):408. doi: 10.3390/jcdd13090408 (PMC13607740; doi:10.3390/jcdd13090408)
Supplement: Supplementary file 1 [file jcdd-13-00408-s001.zip › jcdd-4394630-supplementary.pdf]

Supplementary Material

Table S1. FIB-4 tier × ICD exposure cross-tabulation.

FIB-4 tier	Advanced LD (ICD) n=102	Other CLD (ICD) n=26	No coded LD n=909
Low (<1.45)	22	8	358
Indeterminate (1.45–3.25)	35	11	379
Advanced (>3.25)	43	7	168

Of 909 patients without any coded liver disease, 168 (18.5%) had FIB-4 >3.25, indicating substantial occult advanced fibrosis missed by clinical coding. Conversely, 22 of 102 patients (21.6%) with ICD-coded advanced liver disease had FIB-4 <1.45, possibly reflecting treated/compensated disease or coding without active disease activity.

Table S2. Sensitivity analyses.

Outcome	Exposure n	Exposure %	Reference n	Reference %
S1. Per-protocol cohort: excluding prior MI / PCI / CABG / liver-transplant codes (Advanced LD n=68 vs No LD n=575)				
Invasive management	9	13.2	303	52.7
PCI	3	4.4	195	33.9
In-hospital mortality	30	44.1	48	8.3
Acute kidney injury	45	66.2	121	21.0
Cardiogenic shock	16	23.5	41	7.1
Length of stay, median (days)	12.8	—	3.6	—
S2. Broader exposure: any chronic liver disease vs no LD (any CLD n=128 vs No LD n=909)				
Invasive management	24	18.8	458	50.4
PCI	13	10.2	291	32.0
In-hospital mortality	45	35.2	72	7.9
Acute kidney injury	76	59.4	211	23.2
Length of stay, median (days)	11.2	—	3.9	—

S1 (per-protocol cohort) excludes patients carrying ICD/CPT codes for prior MI (I25.2), prior PCI (Z95.5), prior CABG (Z95.1), or prior liver transplant (Z94.4) at any prior encounter. S2 (broader exposure) replaces ICD-defined Advanced LD with the union of advanced LD plus fatty liver, viral hepatitis, and other unspecified chronic liver disease codes. Both sensitivity analyses preserved the direction and significance of all primary findings. The per-protocol cohort comprises 662 patients in total (Advanced LD n=68, No LD n=575, and Other CLD n=19, retained in the cohort but not displayed in S1 to focus on the primary comparison); the 375 excluded patients comprise 34 Advanced LD, 334 No LD, and 7 Other CLD patients.

Table S3. Sensitivity analysis: mortality and AKI models additionally adjusted for cardiogenic shock (ICD-based exposure, n=1011).

Model	Primary (no shock)	Sensitivity (with shock)	Attenuation
In-hospital mortality, primary multivariable model	8.22 (5.02–13.46)	6.77 (4.06–11.29)	–18%
In-hospital mortality, additionally adjusted for invasive management	5.89 (3.55–9.77)	4.85 (2.85–8.24)	–18%
Acute kidney injury	5.91 (3.63–9.62)	5.09 (3.09–8.36)	–14%

Values are adjusted odds ratios (95% CI) for Advanced LD vs No LD. Primary models adjust for age, sex, diabetes, CKD, heart failure, and ST-elevation (AKI model omits ST-elevation); sensitivity models additionally include cardiogenic shock as a covariate. Attenuation indicates the proportional reduction in the aOR when shock is added to the model. All P-values remain <0.001 in both columns. The primary models estimate the total association between liver disease and each outcome; because cardiogenic shock may lie on the pathway between liver disease and death, and because its timing could not be reliably established in our source data, it was not included in the primary models. The sensitivity column describes the portion of the association remaining after acute presentation severity is additionally accounted for.
